# Stance and Swing Detection Based on the Angular Velocity of Lower Limb Segments During Walking

**DOI:** 10.3389/fnbot.2019.00057

**Published:** 2019-07-24

**Authors:** Martin Grimmer, Kai Schmidt, Jaime E. Duarte, Lukas Neuner, Gleb Koginov, Robert Riener

**Affiliations:** ^1^Lauflabor Locomotion Laboratory, Department of Human Sciences, Institute of Sports Science, Technische Universität Darmstadt, Darmstadt, Germany; ^2^Sensory-Motor Systems (SMS) Lab, Department of Health Sciences and Technology, Institute of Robotics and Intelligent Systems (IRIS), ETH Zurich, Zurich, Switzerland; ^3^Spinal Cord Injury Center, University Hospital Balgrist, Zurich, Switzerland

**Keywords:** walking, stance, swing, inertial measurement unit, gait phase, detection, control, exoskeleton

## Abstract

Lower limb exoskeletons require the correct support magnitude and timing to achieve user assistance. This study evaluated whether the sign of the angular velocity of lower limb segments can be used to determine the timing of the stance and the swing phase during walking. We assumed that stance phase is characterized by a positive, swing phase by a negative angular velocity. Thus, the transitions can be used to also identify heel-strike and toe-off. Thirteen subjects without gait impairments walked on a treadmill at speeds between 0.5 and 2.1 m/s on level ground and inclinations between −10 and +10°. Kinematic and kinetic data was measured simultaneously from an optical motion capture system, force plates, and five inertial measurement units (IMUs). These recordings were used to compute the angular velocities of four lower limb segments: two biological (thigh, shank) and two virtual that were geometrical projections of the biological segments (virtual leg, virtual extended leg). We analyzed the reliability (two sign changes of the angular velocity per stride) and the accuracy (offset in timing between sign change and ground reaction force based timing) of the virtual and biological segments for detecting the gait phases stance and swing. The motion capture data revealed that virtual limb segments seem superior to the biological limb segments in the reliability of stance and swing detection. However, increased signal noise when using the IMUs required additional rule sets for reliable stance and swing detection. With IMUs, the biological shank segment had the least variability in accuracy. The IMU-based heel-strike events of the shank and both virtual segment were slightly early (3.3–4.8% of the gait cycle) compared to the ground reaction force-based timing. Toe-off event timing showed more variability (9.0% too early to 7.3% too late) between the segments and changed with walking speed. The results show that the detection of the heel-strike, and thus stance phase, based on IMU angular velocity is possible for different segments when additional rule sets are included. Further work is required to improve the timing accuracy for the toe-off detection (swing).

## 1. Introduction

Exoskeletons can be used by people with mobility impairments to assist during rehabilitation (Kilicarslan et al., 2013; Arun Jayaraman and Rymer, 2017) or to provide assistance in their everyday life (Quintero et al., 2011; Esquenazi et al., 2012; Strickland, 2012; Aach et al., 2014; Awad et al., 2017; Schmidt et al., 2017; Grimmer et al., 2019b). One of the primary movements that lower limb exoskeletons assist with is walking. Walking is a cyclic process that is comprised of multiple reoccurring patterns to move in space while maintaining static and dynamic balance (Winter, 2009). One cycle of walking, also known as stride, is defined as a movement sequence beginning and ending with consecutive heel-strikes of the same foot. A separation of the stride into the stance phase and the swing phase is widely used to differentiate when the foot is in contact with the ground and when the limb is swinging forward (Taborri et al., 2016). A proper characterization of the walking phases is crucial for lower limb exoskeletons since incorrect timing can increase user effort and the risk of stumbling or falling. In laboratory-based gait analysis, the stance and the swing phase are typically determined by using the vertical ground reaction force, which is measured with force plates (Hendershot et al., 2016). If not available, also motion capture data can be used (Hendershot et al., 2016). This work will evaluate a novel concept for stance and swing detection that can be used to control autonomous wearable robots, such as lower limb exoskeletons in non-laboratory conditions.

Lower limb exoskeletons rely on sensors to detect the gait phases during walking. Pressure insoles (Preece et al., 2011; Lee et al., 2014; Yu et al., 2015) and foot switches (Skelly and Chizeck, 2001; Bae and Tomizuka, 2011; Agostini et al., 2014) are commonly used to detect stance and swing phase because they provide reliable signals of the ground contact. Signals from accelerometers (Mansfield and Lyons, 2003; Selles et al., 2005; Sant'Anna and Wickström, 2010), gyroscopes (Tong and Granat, 1999; Catalfamo et al., 2010; Mannini and Sabatini, 2011; Formento et al., 2014; Gouwanda and Gopalai, 2015), and electromyography (EMG) sensors (Lauer et al., 2004; Kawamoto et al., 2010; Joshi et al., 2013) can also supply signals for gait segmentation. Capacitive sensors (Zheng et al., 2017) and ultrasonic sensor arrays (Qi et al., 2016) can also be used to measure the shape of the user's muscles when the limb is inside of a cuff, but they are less common due to their increased complexity. In a typical use case, sensor signals are fused together to create a high-quality aggregate signal by reducing the uncertainty compared to when one sensor is used alone. A common example of fusing sensor signals to segment gait is the use of inertial measurement units (IMUs) (Hanlon and Anderson, 2009; Liu et al., 2009; Schmidt et al., 2017), which consist of accelerometers, gyroscope, and magnetometers. The combination of the sensors allows to determine a drift free absolute angle of a segment in space. Another approach is to combine gyroscopes with pressure insoles (Pappas et al., 2004) or gyroscopes with force sensors (Asbeck et al., 2015). While the gyroscopes provide kinematic inputs that can also be used to identify sub-phases within swing phase, the pressure and force sensors provide kinetic inputs that primarily are used to identify stance and swing phase, and to separate sub-phases during stance.

The algorithms for gait phase detection usually consist of state-machines with simple thresholding and peak detection (Tong and Granat, 1999; Pappas et al., 2004; Liu et al., 2009; Asbeck et al., 2015), or with more advanced techniques, such as oscillator-based algorithms (Yan et al., 2017), Fuzzy-logic (Kong and Tomizuka, 2008; Alaqtash et al., 2011), Bayesian inference (Malešević et al., 2014), Hidden-Markov models (Bae and Tomizuka, 2011; Mannini and Sabatini, 2011; Taborri et al., 2014; Mannini et al., 2015), or neural networks (Jung et al., 2015; Liu et al., 2016). Advanced algorithms tend to be more versatile for detecting different walking patterns and conditions. However, it is challenging to implement these advanced algorithms in microcontrollers. While state machines are deterministic systems where all possible outcomes can be examined, more advanced algorithms often rely on probabilistic outputs. The certification of devices using probabilistic algorithms is difficult since, the state of a system cannot be known at all times. This article focuses on the suitable implementation for deterministic state machines.

**Figure 1:**
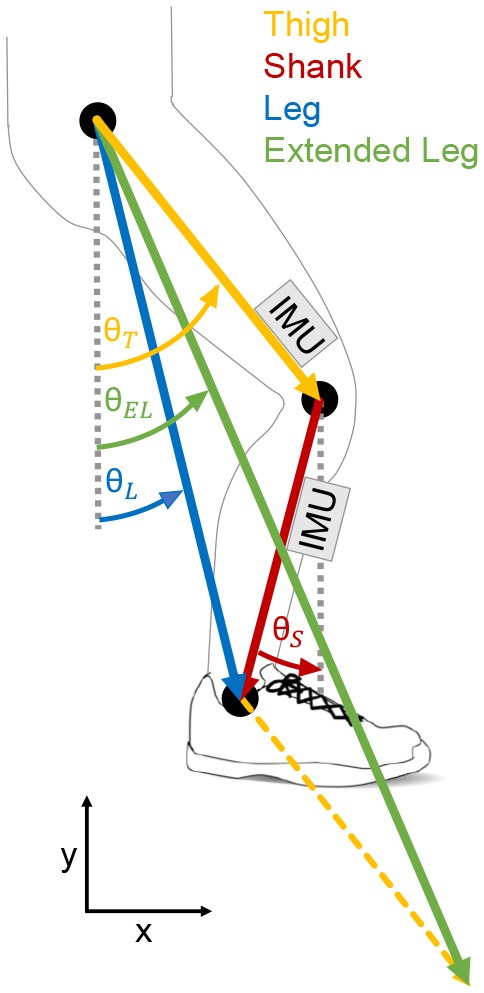
Biological and virtual lower limb segments evaluated for the potential on stance and swing detection. The evaluated biological segments are the thigh (orange) and the shank (red). The evaluated virtual segments (based on Villarreal and Gregg, 2014) are the leg (blue) and the extended leg (green). While the virtual leg is a combination of thigh and shank, the virtual extended leg is a combination of the virtual leg and the thigh. Inertial measurement units (IMU) were place at the thigh and the shank to determine the segment angles and the segment angular velocities.

Gait phase segmentation based on the kinematics of biological lower limb segments (thigh, shank, foot) has achieved promising results in previous experiments (Tong and Granat, 1999; Liu et al., 2009). However, Villarreal and Gregg extended the concept of using the kinematics of these segments alone by creating virtual limb segments of the lower limb (Figure 1) based on geometrical projections of the thigh and the shank (Villarreal and Gregg, 2014). Such virtual legs are also used in conceptual gait models, which are able to represent lower limb dynamics (Geyer et al., 2006). A virtual leg was created by combining the shank and the thigh vector. An extended virtual leg was created by additionally adding the thigh vector to the virtual leg vector. Villarreal and Gregg used the angle and the angular velocity of the virtual lower limb segments to create a phase angle (Holgate et al., 2009), which can be used to determine the progress of each stride (gait percent) independent of time. The phase angle of the virtual segments showed improved monotonic behavior during stance and swing phase compared to the biological segments. For different walking and running speeds as well as stair ascending and descending the best monotony was identified for the extended virtual leg. With improved monotony, there are less changes in the angular direction of a limb segment angle and thus less changes in sign (zero crossing) of the corresponding angular velocity.

However, we believe that the angular velocities of these virtual limb segments also have great potential to be used for the determination of the gait phases. The angular velocity could be determined by IMUs at the lower limb. Compared to approaches including pressure insoles or force sensors such an approach could simplify the portable sensor setup. We believe that the angular velocity of a lower limb segment could be a good variable to distinguish stance and swing, as a comparable concept can be found in a simplified human walking model, the bipedal spring-mass model (Geyer et al., 2006). It incorporates a stance phase, where the leg of the model rotates backwards, starting with the heel-strike. After toe-off the leg is reset (forward rotation) to the angle required for the following heel-strike.

**Figure 2:**
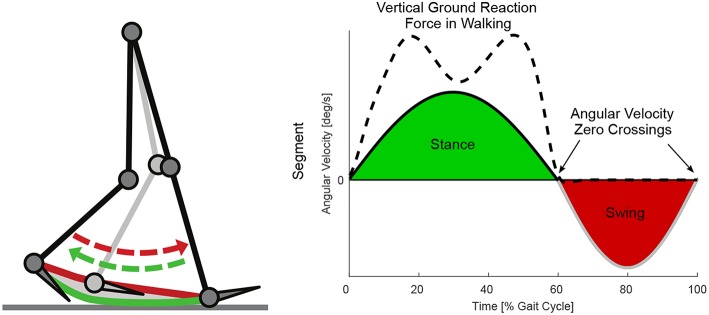
Lower limb segment velocity based concept for stance and swing detection. The shown angular velocity profile represents a conceived example case with similar timing of the angular velocity zero crossings from negative to positive at heel-strike, and the similar timing of the angular velocity zero crossings from positive to negative at toe-off. Heel-strike and toe-off can be determined by the existence vertical ground reaction force.

Based on this concept, we hypothesize that the sign of the angular velocity of lower limb segments, as shown in Figure 2, can be used to identify stance and swing phase during walking at different speeds and inclinations. In addition, we hypothesize that the timing of the sign changes could be used to identify the events heel-strike and toe-off. As the foot segment has no angular velocity during most of the stance phase (Jasiewicz et al., 2006; Bae et al., 2018) and it was not planned to use a foot IMU in the Myosuit (Schmidt et al., 2017), it was not analyzed in this study.

We aim to evaluate the performance of sign-based angular velocity gait phase segmentation for biological and virtual lower limb segments. Specifically, we are interested in the reliability of this approach to detect stance and swing, and in the accuracy in the detection of heel-strike and toe-off. In this context, reliability refers to the ability of detecting only one swing and one stance phase per stride, and accuracy refers to the ability of detecting heel-strike and toe-off events at the right timing.

We first analyzed the utility of both biological (shank, thigh) and both virtual (leg, extended leg) lower limb segments, for stance and swing detection. Therefor we determined the sign changes of the segment angular velocities based on optical motion capture data and data from portable IMUs. The inclusion of both will help to separate gait specific effects and IMU specific effects. A suitable segment should only show two angular velocity zero crossings: one near heel-strike from negative to positive and one near toe-off from positive to negative (Figure 2). To evaluate and compare their timing accuracy regarding heel-strike and toe-off, the timing of the single sign changes of the IMU based and Motion capture based angular velocities will be compared to the timing of vertical ground reaction forces determined by an instrumented treadmill. As the Motion capture data won't be available for an autonomous exoskeleton like the Myosuit (Schmidt et al., 2017), the focus will be on the IMU data.

## 2. Materials and Methods

### 2.1. Study Protocol

This study recorded and analyzed walking kinematics and kinetics of 13 subjects (five females and eight males, age 26 ± 4 years, height 178 ± 11 cm, weight 69.5 ± 11.5 kg) without gait impairments. The study protocol was approved by the institutional review board of ETH Zurich. All subjects gave written informed consent in accordance with the Declaration of Helsinki. Subjects walked on various ground inclinations and at various walking speeds on an instrumented split-belt treadmill. Walking speeds during level walking were 0.5, 0.9, 1.3, 1.7, and 2.1 m/s. Walking speeds during decline and incline walking were 0.9, 1.3, and 1.7 m/s. The decline and incline angles where −10, −5, +5, and +10°.

**Figure 3:**
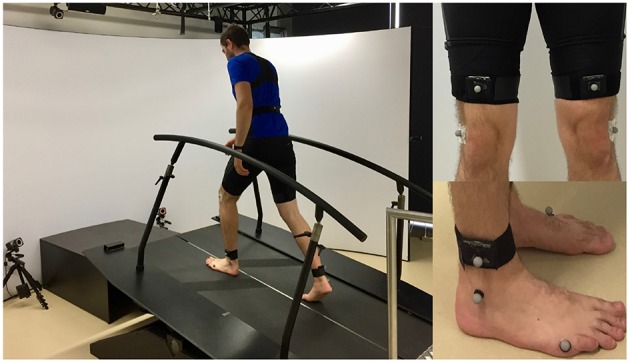
Measurement setup including an instrumented split-belt treadmill and a motion capture system **(left)**. Inertial measurement unit (IMU) setup for the thigh and the shank **(right)**. The sensor units were placed in textile straps. The length of the straps was adapted to differences in body composition using velcro. Reflective markers on the sensors were not used for this evaluation.

Ground reaction forces (GRF) were recorded using the instrumented treadmill (Figure 3, R-Mill—also known as GRAIL, Motek Medical B.V., motekforcelink.com, Netherlands). The kinematics of the lower limb segments were recorded simultaneously using a ten camera motion capture system (Bonita B10, Vicon Motion Systems, UK) and a set of five inertial measurement units (9-axis IMUs; MPU-9250, TDK, Japan). The IMUs (weight 0.028 kg, range accelerometer ±8 g, range gyroscope 500 °/s) were used at a sampling rate of 133 Hz. They were packaged in the SimpleLink SensorTag (CC2650STK, Texas Instruments, USA) and Bluetooth Low Energy was used to transmit the IMU data to a Single Board Computer (RPi3, Raspberry Pi Foundation, UK). All systems were synchronized before the data collection.

We recorded 1 min of continuous walking for each walking speed, starting with the slowest speed. Subjects first walked on level ground, then uphill (5 and 10°), and finally downhill (5 and 10°). The protocol required subjects to walk continuously at each inclination condition. A rest period of 5 min was required between the level, uphill, and downhill walking trials to store the data and to restart the measurement systems. Breaks of <1 min were required to change between the uphill and downhill slopes of 5° and 10°.

In this article, we focused on the greatest slope (10°), and the speeds 0.5, 1.3, and 2.1 m/s for level walking to reduce the amount of data. The outcomes of the omitted data are in line with the presented results. All calculations were performed offline.

### 2.2. Evaluation of Kinematics and Ground Reaction Forces

Three-dimensional ground reaction forces were recorded at 1,000 Hz. The vertical GRF was used to determine the reference times for the heel-strike and toe-off of each stride. A vertical GRF >400 N was used to initiate the identification of the heel-strike event. A value of 400 N was selected based on experience from previous studies to avoid heel-strike detection's during swing, when the opposite leg was slightly touching the wrong belt of the split-belt treadmill. From that data point, an algorithm scanned backwards in time to detect the first sample that was <0 N or that was greater than the subsequent force value and <10 N. Values smaller than 0 N were observed due to the vertical GRF signal noise that oscillates around 0 N with an amplitude of <10 N. The second condition with the 10 N limit was selected to increase the accuracy of the heel-strike detection for the case when a positive part of the oscillation was directly before the heel-strike. To determine the toe-off time, the GRF was filtered with a zero-lag, second-order, low-pass Butterworth filter (cutoff frequency 20 Hz). It was filtered to eliminate small GRF oscillations due to the heel-strike that would have influenced the correct detection of the toe-off. Beginning 100 ms after each heel-strike, an algorithm identified the toe-off time based on the first subsequent GRF that was <10 N.

Body segment kinematics were recorded at 200 Hz using reflective markers from the motion capture system. The marker data was up-sampled linearly to 1,000 Hz to match the frequency of the ground reaction forces. Raw marker data was filtered with a zero lag, fourth-order, low-pass Butterworth filter (cutoff frequency 10 Hz). The thigh and shank angles and angular velocities were filtered using this same method. The thigh segment was determined by a marker at each hip (greater trochanter head) and each knee (2 cm proximal of the joint space on the lateral femoral condyle). The shank angle was determined by the knee marker and an ankle marker (lateral malleolus). The leg angle and extended leg angle were calculated as described in Villarreal and Gregg (2014) (see also Figure 1). The angular velocities of the leg and extended leg segment were determined by numerical differentiation.

The kinematics of the thigh and the shank of each limb were recorded using IMUs (Figure 3). Thigh and shank angles were determined during post-processing with Matlab by combining gyro and accelerations signals using a Kalman filter (process noise covariance = 0.00005; measurement noise covariance = 7). When calculating the virtual leg and the virtual extended leg angle (Villarreal and Gregg, 2014), thigh and shank length were assumed to be equal (24.5% body height, based on Winter, 2009). We believed that both virtual segments are less dependent on the individual biological segment movement and thus they might provide a more general segment angular velocity profile with a single zero crossing per direction for the variety of tested walking speeds and slopes. Thus, the improved monotony that was shown for such virtual segments by Villarreal and Gregg (2014) would be beneficial for our concept to distinguish stance and swing (Figure 2). Villarreal and Gregg did not describe why next to the virtual leg (sum of thigh and shank vector) the virtual extended leg (sum of two times the thigh and a single shank vector) was investigated. When exploring their results, we found that the monotony of stair ascent was most critical. The double influence of the thigh increased the overall range of motion of the extended leg angle during stair ascend, which improved the monotony. Further, when using the extended leg, the range of motion for stair descent was decreasing. Based on both findings we assume that there could be an optimal scaling of the thigh and the shank proportion based on the analyzed gait. We did not investigate on the optimal proportion in this work.

The average gait data of each subject (subject condition average) was determined based on 65 to 159 strides (sum of both limbs) for each condition of slope and speed. Afterwards, each subject condition average was aggregated to obtain the subject group condition average and the corresponding standard deviation. The data included in this work is based on 4,377 strides for level walking, 3,579 strides for walking uphill, and 4,082 strides for walking downhill.

### 2.3. Sign Changes of the Angular Velocity

The reliability of each lower limb segment was calculated by aggregating all zero crossings of the angular velocity per stride. Since sign changes are to be used to indicate the transition between stance and swing phases, only one transition of the angular velocity from positive to negative and one from negative to positive were biomechanically relevant (Figure 2). Additional transitions, that do not match our concept, resulted in higher counts (mean value more than one) of zero crossings. The most reliable signal would be the one with the least amount of transitions.

Further, the accuracy of the transitions was evaluated. Therefor each transition timing was analyzed with respect to the corresponding reference event timing of heel-strike or toe-off. As described previously, heel-strike and toe-off timing were determined using the vertical GRF. The time difference to the reference event was calculated as a percentage of the gait cycle. A detection before the reference event results in a negative percentage whereas a detection after the reference event results in a positive percentage.

### 2.4. Rule Sets for Event Detection

**Figure 4:**
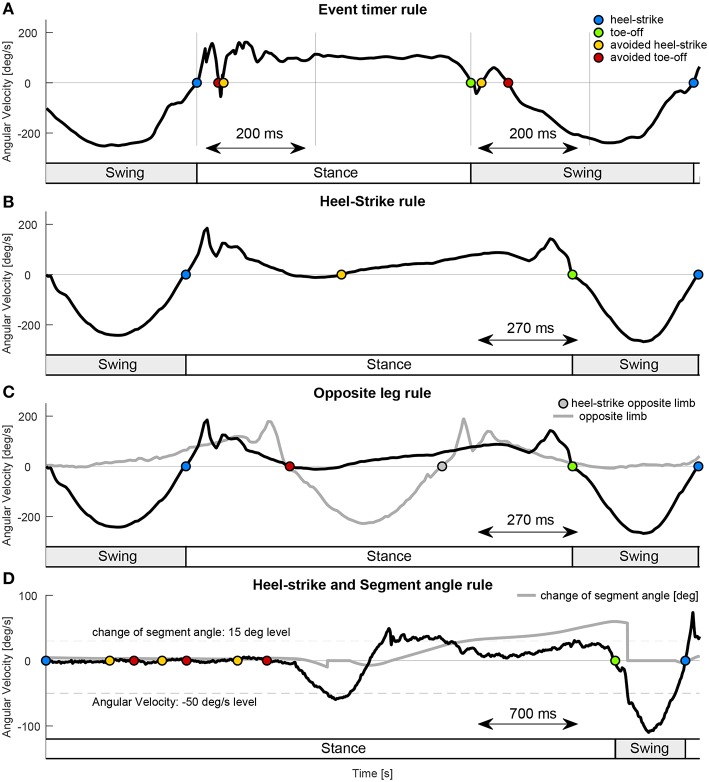
Examples for the applied rule sets. **(A)** The Event timer rule used a defined time interval after each event detection (heel-strike blue, toe-off green) to avoid additional hell-strike (yellow) or toe-off (red) detections. The example used an interval of 200 ms for the virtual leg during walking declines (1.7 m/s). **(B)** The Heel-strike rule ensured that after heel-strike only toe-off and after toe-off only heel-strike can be detected. In this example the Opposite leg rule is required in addition to also avoid the toe-off detection. **(C)** The Opposite leg rule required the heel-strike of the opposite limb (gray solid) before a toe-off can be detected. The example data for **(B,C)** is from the shank segment during walking inclines (0.9 m/s). **(D)** The Segment angle rule (was not applied for the analysis) required a change of 15° in the segment angle (set to zero when angular velocity larger than −50 °/s) to allow toe-off (green) detection. In combination with the Heel-strike rule it can avoid toe-off detection while standing. The example data is from the shank segment during gait initiation of walking inclines. Heel-strike is set as initial condition. The timing of the stance and the swing, indicated by the bottom bars for **(A–D)** is based on the rule sets.

To analyze the accuracy of the transitions it is required that each lower limb segment only detects one transition in angular velocity from negative to positive and one from positive to negative (Figure 2). As this was not the case for the IMU data and some of the motion capture data improved methods were required to be able to use the segment angular velocity for stance and swing detection. Detection rule sets were designed (Figure 4) to detect just one transition in angular velocity for each direction, and to increase the likelihood of detecting transitions near to the timing of the GRF based events. Temporal and causal relations, as well as absolute thresholds were used, hand tuned, and tailored individually to each lower limb segment (thigh, shank, leg, extended leg). To define the rule sets for each lower limb segment, only the biomechanics of the same segment were used. A transition of the angular velocity from positive to negative or vice versa served as the main trigger before any of the detection rule sets were applied. Unfortunately, we were not able to reduce the amount of transitions to just two transitions per stride (near to the real events) for all speed and slope condition of the thigh segment. Thus, we had to excluded the thigh segment from the analysis of the timing accuracy.

Blocking times after an event detection (Event Timer rule, Table 1, Figure 4A) were used to prevent multiple detections (both heel-strike and toe-off) within a given time window. The constraints used were: 150 ms for the shank, 200 ms for the leg, and 250 ms for the extended leg segment. Increased time periods were mainly required to overcome multiple detections for the leg and extended leg at toe-off. For simplification, just one time value was selected for each segment. For safety reasons, we tried to keep these values as small as possible. This allows a heel-strike, in case of stumbling, as soon as possible.

**Table 1:** Rule sets applied to the different lower limb segments.

	**Event**	**Heel-strike**	**Opposite leg**
	**timer rule**	**rule**	**rule**
Thigh*	–	–	–
Shank	150 ms	Used	Used
Leg	200 ms	Used	Used
Extended leg	250 ms	Used	–

While we were able to allow only one transition from negative to positive angular velocity and one in the other direction for the shank, leg, and extended leg segment, we were not able to limit transitions with the same rule sets for the thigh segment. Thus, the thigh* was excluded from the analysis of the accuracy (on-time detection).

An additional rule ensured that by following a heel-strike event, only toe-off can be identified, and vice versa (Heel-strike rule, Table 1, Figure 4B). This rule prevented the consecutive detection of the same events. As by nature, a zero crossing from one to the other direction will always be followed by the opposite version, during walking this rule was only applied in the rare cases where the first wrong zero crossing was eliminated by the Event Timer rule but the corresponding wrong follow up detection to the other direction was not. In addition, it worked together with the Opposite leg rule to eliminate a second heel-strike detection during stance, mainly for the shank segment.

Another rule was based on the opposite leg, such that heel-strike of the opposite leg must precede toe-off (Opposite leg rule, Table 1, Figure 4C). This condition ensured that one leg was always in stance. This rule relied on the detection of double-support when both legs are on the ground. This rule was especially helpful for the shank segment as the zero crossing (positive to negative) during midstance was eliminated from detection. The rule could not be applied for the extended leg segment, as for some strides the toe-off was detected before the heel-strike of the opposite leg.

An additional rule set, to improve detection reliability, was based on the absolute change of the segment angle (Segment angle rule, Table 1, Figure 4D) during the stance phase. This rule was fundamental to avoid a detection of toe-off due to little lower limb oscillations while standing at the beginning and the end of each trial. It was not required for our analysis as only constant walking velocities were analyzed in this work. As we believe it is an essential rule for the application of our concepts we decided to also explain it at this point. The change in the segment angle was measured with respect to the segment angle during late swing, when the angular velocity became >−50 °/s. A change of 15 ° would enabled the detection of the toe-off event for all analyzed segments. The threshold of the trigger was set to −50 °/s (for all segments), rather than 0 °/s, to improve detection, as the value is higher than found in the oscillations during standing. When the gait phase is initially set to standing, both the –50 °/s of the segment angular velocity and the consecutive change of 15 ° are required to enable the first and all following swing phases. For some rare cases, it can also prevented from early toe-off detections (leg and extended leg) during late stance.

## 3. Results

### 3.1. Temporal Progression of the Angular Velocity

**Figure 5:**
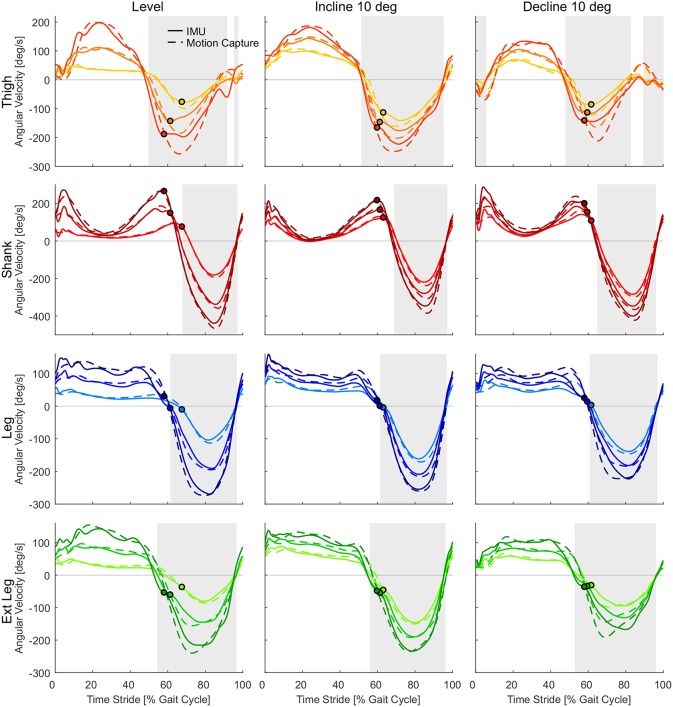
Angular velocities from inertial measurement units (solid) and motion capture (dashed) for the thigh (orange), shank (red), leg (blue), and extended leg segment (green) in level walking, inclined walking (10°) and declined walking (10°). Darker colors indicate higher speeds (0.9, 1.3, and 1.7 m/s for slopes, 0.5, 1.3, and 2.1 m/s for level walking). Circles indicate the related toe-off determined by the vertical ground reaction force. The heel-strike and the following heel-strike of the same limb occur at 0 and 100% of the stride time. Gray areas indicate the swing phase based on the angular velocity of the IMU at 1.3 m/s.

**Figure 6:**
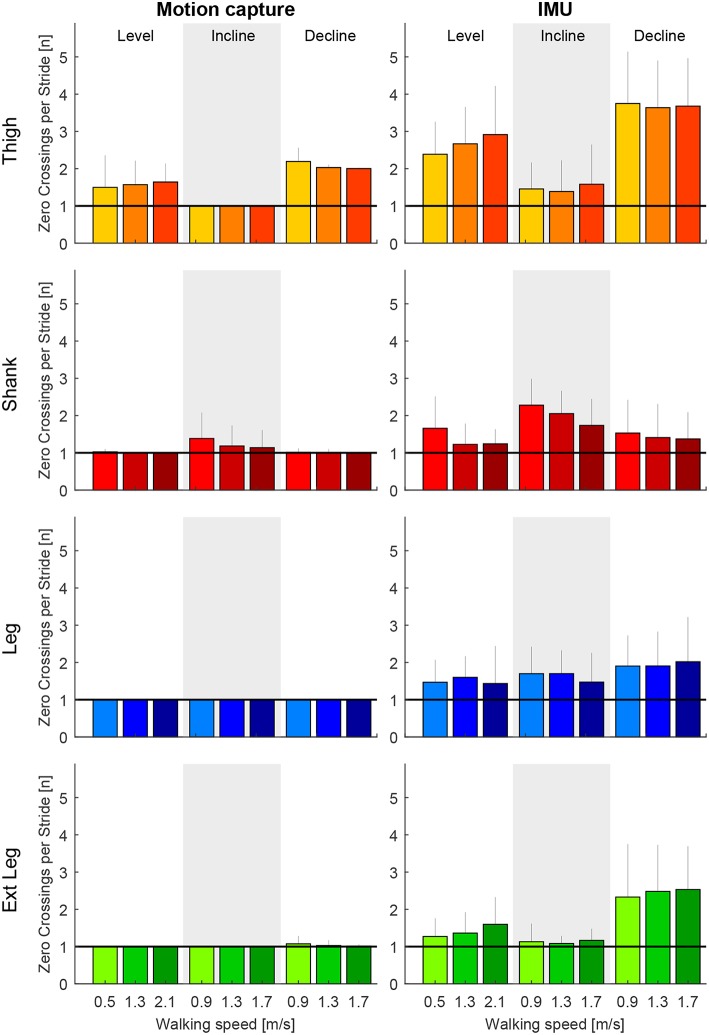
Number of zero crossings of the angular velocity per gait cycle for the thigh (orange), shank (red), leg (blue), and extended leg segment (green) in level walking, inclined walking (10°) and declined walking (10°). The angular velocity was determined by motion capture **(left)** and by inertial measurement units **(right)**. Darker colors represent greater speeds (0.9, 1.3, and 1.7 m/s for slopes, 0.5, 1.3, and 2.1 m/s for level walking). Error bars represent one standard deviation.

The subject group means of the angular velocities recorded by the IMUs and the motion capture system were comparable with regard to the shape and magnitude for all lower limb segments (Figure 5). For both measurement methods an increase in walking speed resulted in higher peak angular velocities. We found that all lower limb segments (thigh, shank, leg, and extended leg) showed a positive angular velocity during the first part of the gait cycle and a negative angular velocity in the second part. For the group mean values, sign changes from positive to negative angular velocity occurred between 50 and 70% of the gait cycle considering all speed and slope conditions. The positive to negative transitions for the thigh model occur first in the gait cycle whereas the shank model marks the last transition (see Figure 5). Sign changes from negative to positive occurred between 80 and 10% of the gait cycle for the group means when including all conditions. The thigh segment marked the first and the last negative to positive transitions in the gait cycle due to multiple zero crossings. It has to be noted that the subject group means will hide individual gait characteristics including additional sign changes. The total amount of sign changes for each segment can be found in Figure 6.

### 3.2. Reliability of Segment Angular Velocity for Gait Phase Detection

**Figure 7:**
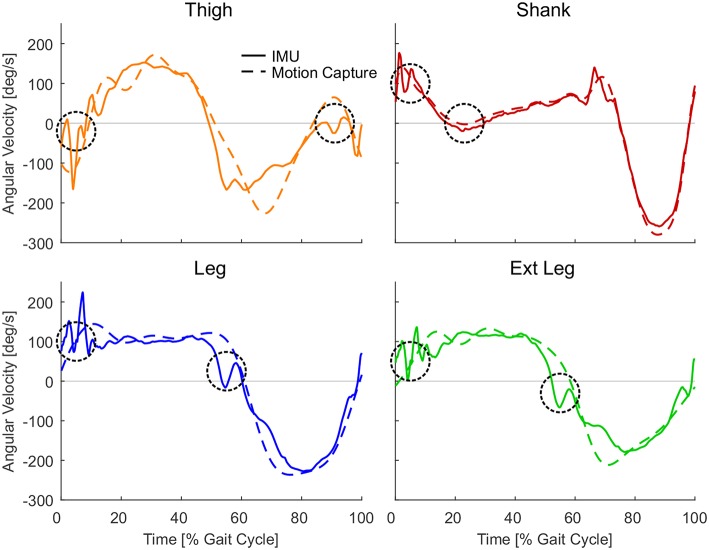
Single subject angular velocities for one stride from the inertial measurement units (solid) and the motion capture (dashed) for the thigh (orange, descent −10° at 1.7 m/s), shank (red, ascent 10° at 0.9 m/s), leg (blue, descent −10° at 1.7 m/s), and extended leg (green, descent −10° at 1.7 m/s) segment. Circles indicate phases where the IMU data can have unwanted sign changes in the angular velocity due to oscillations or by the nature of the segment movement. Representative strides were selected from one subject and condition with the greatest number of zero crossings shown in Figure 6.

The thigh angular velocity had multiple zero crossings before and after heel-strike for level walking and when walking at declines (Figure 5). The shank angular velocity showed a local minimum close to zero during mid-stance when walking inclinations (Figure 5). The profile of the grand means suggest that the thigh and shank segment are not able to fully describe the anticipated concept shown in Figure 2. This is also reflected when evaluating the total amount of zero crossings (Figure 6) and when looking at individual subject curves (Figure 7).

When comparing both measurement methods, motion capture to IMUs (Figure 6), the filtered motion capture data resulted in the fewest number of angular velocity sign changes per stride. All four lower limb segments had a greater number of angular velocity sign changes when determined by the IMU data. Other than the grand means (Figure 5), individual single stride IMU data (Figure 7) could reveal signal details that resulted in the increased amount of angular velocity sign changes. While the fewest number of angular velocity sign changes were identified for the motion capture data of the leg and the extended leg, both showed oscillation around zero in several subjects and conditions at about 60% of the gait cycle when using the IMU data.

### 3.3. Accuracy of the Angular Velocity Transition Timing

Based on our concept shown in Figure 2, only one sign change of angular velocity should occur at heel-strike and one at toe-off. As multiple sign changes occurred for the IMU data of all lower limb segments, rule sets were introduced to limit the detections to just one per velocity direction and stride. Similar rule sets were also applied to analyze the Motion capture data. Due to multiple zero crossings of the thigh segment in each stride (Figure 5), we were not able to limit the amount of detections with our rule sets as desired. Consequently, we were not able to compare the timing of the sign changes of thigh angular velocity with the timing based on the vertical GRF, however, this comparison was possible for the shank, the leg, and the extended leg segments.

**Figure 8:**
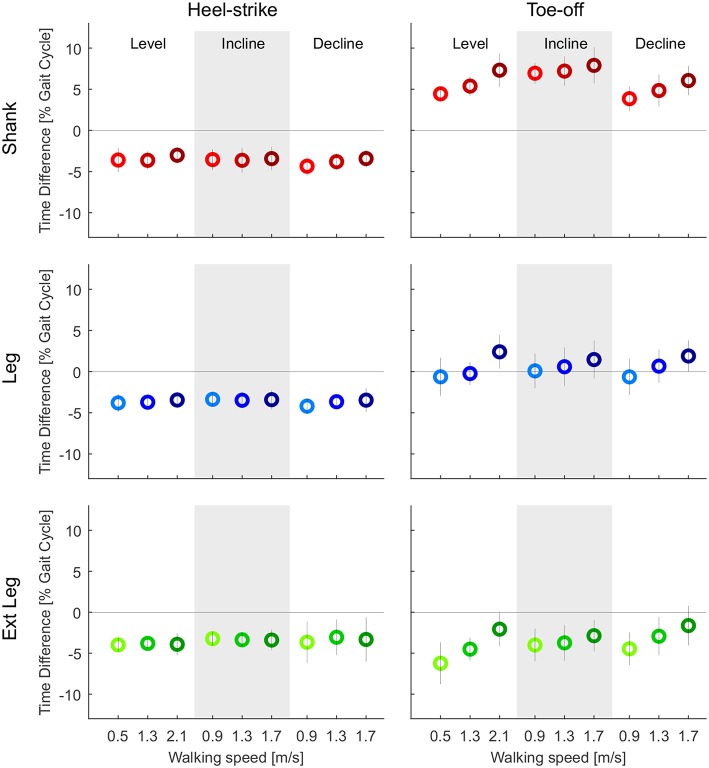
Time difference of the mean zero crossings of the angular velocity based on the optical motion capture data to the heel-strike **(left)** and the toe-off **(right)** identified by ground reaction forces (GRF). Evaluated segment angular velocities from the thigh (orange), shank (red), leg (blue), and extended leg (green). Distances are evaluated for three different speeds of level walking, walking inclines, and walking declines. Darker colors indicate greater speeds (0.9, 1.3, and 1.7 m/s for slopes, and 0.5, 1.3, and 2.1 m/s for level walking). The standard deviation is indicated by the vertical line. The specific detection rule sets, that were designed for the IMU data, were also applied to the Motion capture data to only detect one transition for each event. Positive and negative values indicate zero crossings after and before, respectively, the GRF based event detection.

**Figure 9:**
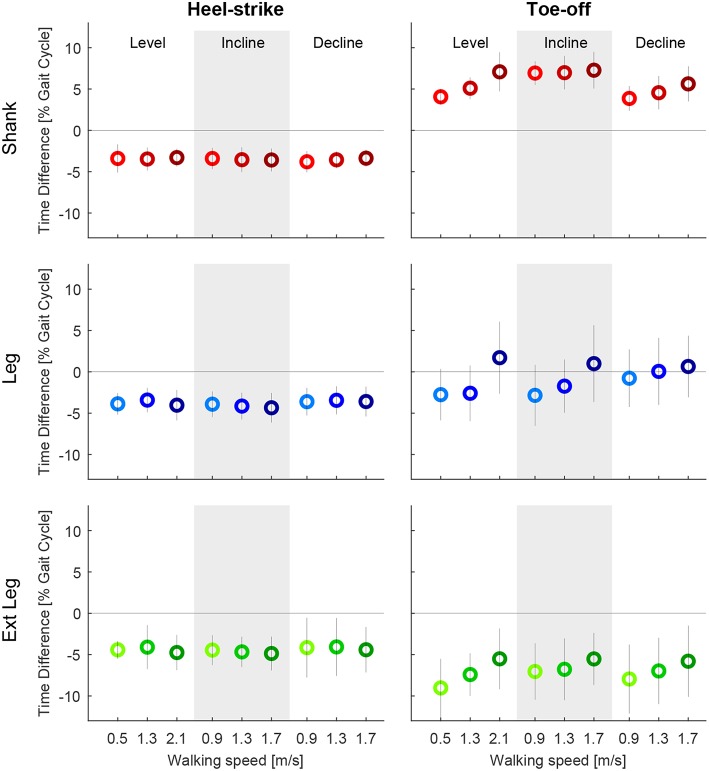
Time difference of the mean zero crossings of the angular velocity based on the inertial measurement unit to the heel-strike **(left)** and the toe-off **(right)** identified by ground reaction forces (GRF). Evaluated segment angular velocities from the thigh (orange), shank (red), leg (blue), and extended leg (green). Distances are evaluated for three different speeds of level walking, walking inclines, and walking declines. Darker colors indicate greater speeds (0.9, 1.3, and 1.7 m/s for slopes, and 0.5, 1.3, and 2.1 m/s for level walking). The standard deviation is indicated by the vertical line. As multiple zero crossings occur for all segments, specific detection rule sets were designed to only detect one transition for each event. Positive and negative values indicate zero crossings after and before, respectively, the GRF based event detection.

Motion capture-based zero crossings of the shank, leg, and extended leg segments occurred 3.0–4.4% of gait cycle earlier than the heel-strike event based on GRF. The IMU-based zero crossings were in a similar range (3.3–4.8%, Figure 8). The average standard deviation (IMU) for all walking conditions was smallest for the shank, compared to the leg and extended leg (0.6% compared to 0.8 and 1.2%, Figure 9). Smaller average standard deviations, compared to the IMU-based data, were achieved for the Motion capture data (shank 0.6%, leg 0.5%, extended leg 0.7%). In contrast, the timing for the toe-off zero crossings varied with respect to the GRF based timing in both directions for all three segments. Similar ranges were found for Motion capture (Figure 8) and IMU (Figure 9) data. For the IMU data of the shank segment it occurred 4.1–7.3% after the GRF based toe-off (Motion capture: 3.8–7.9% after), for the leg from 2.8% before and up to 1.7% after the GRF based toe-off (Motion capture: 0.6% before to 2.4% after), and for the extended leg from about 9.0 to 5.5% before the GRF based toe-off (Motion capture: 6.2% to 1.6% before). Similar to the IMU data for the heel-strike, the shank segment showed the smallest standard deviation for the zero crossing timing at toe-off (0.9%) compared to the leg (1.9%) and extended leg (1.8%). Compared to the IMU, Motion capture data showed reduced average standard deviations (shank 0.8%, leg 1.0%, extended leg 1.0%). While an increase in walking speed had no effect on the timing of zero crossings at heel-strike, it did have an effect on toe-off. We found a temporal shift to later in the gait cycle for all but one shank condition when increasing walking speed. For uphill walking the timing of the shank zero crossing was almost constant at a level comparable to the highest walking speed from level walking. No further walking speed or inclination related effects were identified.

## 4. Discussion

Four different lower limb segments (thigh, shank, leg, and extended leg) were evaluated to assess their reliability in detecting stance and swing phase in the gait cycle, and to determine their accuracy of the transition timing between these phases. Our detection approach was based solely on the angular velocities of the biological and virtual lower limb segments during level-ground and sloped walking.

### 4.1. Reliability of Segment Angular Velocity for Gait Phase Detection

All lower limb segments showed similar shape and magnitude for the angular velocity when based on either motion capture data or IMU data. However, the angular velocities were superior in their reliability when using the motion capture data, as evidenced by fewer sign changes. The IMU-based angular velocities generally showed more oscillations in the signal, which in turn resulted in incorrect detections of a heel-strike or toe-off and larger accuracy standard deviations primarily for the leg and extended leg. Independent of the data acquisition approach (IMU or motion capture), the lower limb segments did not have equal reliability in the gait phase detection. The best performance to reliably distinguish the gait phases based on filtered motion capture data was identified for the leg and the extended leg segments, with a preference for the leg segment. These segments showed in almost all cases single zero crossings close to the timing of the biological transition from stance to swing phase, and vice versa. In contrast, the shank and thigh segments showed in the most cases more than one zero crossing, even with the motion capture data, and the performance of these segments becomes worse when using the IMU data. Based on the data we obtained from our IMU setup, the concept of only using the sign of the segment angular velocity for stance and swing detection had to be refused. Additional rule sets were required, and we had to detect the events heel-strike and toe-off to distinguish the phases. As more than one zero-crossing was found for the motion capture data of the thigh (level and decline) and the shank (incline), we believe that these zero-crossings (shank in midstance and thigh before heel-strike) are due to the nature of the human walking biomechanics. In contrast, the authors believe that oscillations after heel-strike, that were found for all segments, were introduced by the mechanical impact of the heel-strike (Figure 7). Further, oscillations at toe-off may be a result of the opposite limb's heel-strike. As both the shank and thigh sensor signals were combined to determine the angular velocities of the leg and the extended leg segment, the signal noise of the shank and thigh IMUs was aggregated. In addition, multiple detections near toe-off are induced by the opposing movement directions of the shank and the thigh that lead to oscillations of the angular velocity in the leg and the extended leg segments.

### 4.2. Accuracy of the Angular Velocity Transition Timing

The reliability assessment showed that with a portable IMU setup, as used in this work, undesired angular velocity sign changes were identified. To analyze the difference in the vertical GRF based heel-strike and toe-off timing, compared to the timing of the relevant angular velocity sign changes (just performed for the IMU based data), additional detection rule sets were needed. There should be only one sign change per direction during each stride. These detection rule sets were hand tuned and applied to the thigh, the shank, the leg and the extended leg. The thigh segment had to be excluded from the timing analysis, as we were not able to define rule sets to limit the sign changes for this segment as desired. The inability was a result of extensive oscillations that caused multiple transitions that were not within close proximity to heel-strike. We concluded that the thigh segment angular velocity is unsuitable for stance and swing phase detection using our approach. However, additional detection rule sets may improve the signal quality and render the thigh a reliable source to differentiate gait phases. When using the additional rule sets for the shank, the leg, and the extended leg, we found a systematic bias of early detection for the heel-strike event (with IMU and Motion capture), which was found to be a typical human gait characteristic in previous work (swing leg retraction; Seyfarth et al., 2003; Poggensee et al., 2014). In comparison, early, on-time and late detection were identified for toe-off. For a wearable robotic system, an on-time or early detection is often important to ensure a force application at the right moment, whereas late detection could cause an unfavorable force application. For example, the Myosuit (Schmidt et al., 2017) could use the early detection at heel-strike to pre-tension the system for the upcoming force application. In direct comparison, the late toe-off detection of the shank segment seems problematic whereas the on-time detection of the toe-off using the leg and the early detection of the extended leg segment seem suitable for a controller of a wearable robot.

The variability in the event detection poses an additional challenge. About double the standard deviation for the detection of the heel-strike and the toe-off was found for the leg and the extended leg, compared to the shank segment. The standard deviation was less for the Motion capture data of the leg and the extend leg. Therefore, we believe that especially the signal of the thigh IMU lead to increased variability. Based on our results, the shank could be favored for the heel-strike detection. In contrast, the leg and the extended leg segment seem to be of advantage regarding the toe-off timing. So far it is unclear which of the factors, timing or variability, is more critical for application.

### 4.3. Improving Detection Methods

Lower limb segment velocity, originating from motion tracking data, can be seen as an indicator that our concept can work reliably to distinguish stance and swing phase of walking. When testing the IMU data we found several critical issues. To ensure a more reliable and accurate detection, the methodological approaches could be changed or improved, and the following paragraphs introduce possible options.

The signal noise of the IMU based measurements may be strongly dependent on the placement and the interconnection to the human body. While markers for the motion capture system were lightweight and placed on the skin on top of bones, the IMUs were placed inside of a pocket on straps on top of the moving muscles and tendons. The increased inertia of the IMUs and the compliant interface to the human body may have caused an increased signal noise. It must be evaluated how fixation to other places, such as the exoskeleton itself, can reduce the sensor noise.

Detection reliability may be also improved by using additional sensors. An IMU at the foot was successfully used in Jasiewicz et al. (2006) to detect the transition from stance to swing at level self-selected walking speed for unimpaired subjects and subjects with incomplete spinal cord injury. The distinct peak before the zero crossing of foot angular velocity during stance served as a feature to improve toe-off detection. It was demonstrated that foot angular velocities and accelerations were more reliable than the shank angular velocity for the subjects with incomplete spinal cord injury. The peak angular velocity of the foot has to be explored in subsequent studies to investigate if it can also improve the detection for different walking speeds as well as at inclines and declines. An IMU at the foot in combination with an IMU at the shank was used in Grimmer et al. (2019a) to calculate the joint angular velocity of the ankle online. It would be of interest if characteristics of joint angular velocities or the joint angles can serve to improve swing detection. The ankle angle was used to improve the toe-off detection for post-stroke patients in Bae et al. (2018).

Alternatively, ground reaction force sensors or foot switches could be used to further improve the detection. Jasiewicz et al. found in their study that foot switches were as accurate as foot sagittal angular velocity or shank sagittal angular velocity to determine the heel-strike and the toe-off. Further, while IMUs are required for the unique controller of assistive devices like the Myosuit (Schmidt et al., 2017), GRF sensors or foot switches would increase the complexity of the system with respect to donning and doffing procedures, washing, or user adaptations. For other systems that do not require IMUs, such sensors could potentially reduce the complexity.

A reduction of the oscillations that caused multiple zero crossings could be achieved by implementing filtering techniques. Since online filtering introduces a delay, it is desirable to use the signal of a lower limb segment that depicts zero crossing before the toe-off occurs. The angular velocity of the extended leg segment has exactly this property. In such a case, the filter would not only prevent high frequency oscillations, but also shift the timing of the signal closer to the actual gait event measured by the GRF. In a preliminary test, a second order Butterworth filter with a cut-off frequency of 4 Hz was applied to the IMU signal of the extended leg. While standard deviations were only reduced slightly, the amount of additional detections was reduced to half.

Other improvements could include changes or additional detection rule sets. The detection rule set that showed a significant improvement in avoiding multiple detections around toe-off made use of the aphasic movement pattern of opposite limbs during walking. Toe-off detections were only possible after heel-strike was detected for the opposite leg. This ensured double support during walking and served as one of the triggers for toe-off. We believe that this rule set in combination with the Segment angle rule (that was introduced but not used), which secures assistance during standing, are essential rules for the application of our concept. We can also imagine that it is possible to only use the heel-strike detection of the opposite limb, to detect the toe-off. By using such a concept, the heel-strike of one site would trigger the hell-strike of the opposite limb. Such a concepts was previously used in the Runbot, a two legged robot with a reflex inspired control scheme, to trigger the initiation of swing of the opposite limb (Manoonpong et al., 2011).

### 4.4. Application of the Angular Velocity Based Concept

The study investigated if it is possible to detect the stance and the swing based on the angular velocity of lower limb segments to find an appropriate candidate for the Myosuit, an exoskeleton that partially assists the user during stance (Schmidt et al., 2017). The latest version of the Myosuit requires two IMUs, one at the shank and one at the thigh, to detect the knee angle and scale the assistance force accordingly. The evaluated IMU based concept would allow to avoid additional GRF sensors or foot switches at the shoe, which would increase the complexity of the exoskeleton. This study was performed with young subjects without physical impairments, while future users of the Myosuit could be young and old individuals with and without physical impairments. Individuals with a physical impairment may show different kinematic lower limb patterns compared to those observed in our study. It has to be evaluated if the angular velocity based concept can be applied to these populations. Post-stroke individuals suffer from increased asymmetries in between the limbs (Bae et al., 2018). So far it was found that asymmetries in transtibial amputee walking do not seem to influence the detection of the heel-strike based on the shank angular velocity zero crossing (Grimmer et al., 2017). Based on the data of Yang et al. (2013), the concept based on the shank angular velocity could also work for post-stroke hemiparetic gait. It is unknown how the timing between the IMU based events and the GRF based events will change for impaired populations. We believe that for the heel-strike detection the time for the swing leg retraction (3.3–4.8% of gait cycle) could be reduced prior to heel strike for those who require increased horizontal braking forces (e.g., when walking down at surfaces, such as sand). Next to the timing itself, the variation of the timing in between strides has to be explored for other populations. As we did not identify walking speed and incline related effects for the heel-strike detection, we expect a higher possibility of variation for the toe-off detection. In addition, the use of the thigh or the shank segment could be a problem for movement tasks that have phases with almost zero segment angular velocity. Such a phase can be found when ascending stairs, where the shank segment has almost no angular velocity following heel-strike (Formento et al., 2014). As shown by Villarreal and Gregg (2014), the extended leg, that represent the combined movement of the thigh and the shank, is less sensible to such phases. Further, the angular velocity based concept may require adaptations if users have very low walking speeds, as found in individuals with incomplete spinal cord injury (Domingo et al., 2007). The segment angle rule, requires 15 ° of segment angle change throughout stance, which already sets a lower limit to the required stride length (about 0.26 m for the leg segment). The segment angle rule also used a segment angular velocity of −50 °/s to enable the angle detection, which sets a lower limit for the required walking speed (about −100 to −200 °/s were achieved at 0.5 m/s, Figure 5). It may be necessary to adapt these hand tuned values based on the population. Next to the population, an active exoskeleton, as well as overground walking, likely affect the kinematics of the lower limbs. Thus, the timing of the events may change compared to those of this study.

Also while facing a lot of unknowns with our approach regarding the use for individuals with gait impairments, we believe that the movement direction of the lower limb, and thus the segment angular velocity, is an essential gait characteristic that can be used to distinguish stance and swing phase within gait. The concept was developed for exoskeleton users with locomotion capabilities to resist gravity within stance. So far it is unknown how a slightly too early or slightly too late detection of heel-strike and toe-off will influence the exoskeleton based assistance. Users may adopt the timing of their own contribution to compensate for missing support during stance phase. This may be no problem for them as long the exoskeleton provides a reliable (similar timing and assistance torque scaling) support throughout stance.

## 5. Conclusion

This study aimed to evaluate the feasibility of reliably detecting stance and swing phase based on the sign change of the angular velocity of biological (thigh, shank) and virtual (leg, extended leg) lower limb segments during walking. In addition, the study analyzed the timing of the sign change, and thus the accuracy of the detection for the transition from stance to swing (toe-off) and from swing to stance (heel-strike). We found that it is required to detect the events of the sign changes (heel-strike and toe-off) to separate stance and swing phase, rather than using the sign of the velocity on its own. When using such a phase detection approach in an online application using IMU-based measurements, a reliable and accurate algorithm requires additional rule sets, based on temporal and causal relations that can be found in walking patterns. For the detection of heel-strike events–and thus the beginning of the stance phase–the shank, the leg, and the extended leg segments were identified to all be good candidates. The most promising segment was the shank as it showed the least variability in the detection timing. For the detection of toe-off events, detection timings changed with increasing speed and variability was rather high for both candidates, the leg and the extended leg, which had an acceptable timing.

Further analyses must look into how sensitive users with and without gait impairments are regarding assistance timing for exoskeletons, such as the Myosuit (Schmidt et al., 2017), which partially support the user during stance. Based on the user group, improved detection methods or adaptations in timing may be required.

## Data Availability

The datasets generated for this study are available on request to the corresponding author.

## Ethics Statement

The study protocol was approved by the institutional review board of ETH Zurich. All subjects gave written informed consent in accordance with the Declaration of Helsinki.

## Author Contributions

The study concept was developed by MG, KS, LN, and GK. LN and GK performed the experiments. Data analysis was performed by MG. Interpretation was performed by all authors. MG, KS, and JD were responsible for drafting the article. All authors revised the article and provided approval for publication of the content and agreed to be accountable for all aspects of the work in ensuring that questions related to the accuracy or integrity of any part of the work are appropriately investigated and resolved.

### Conflict of Interest Statement

The authors declare that the research was conducted in the absence of any commercial or financial relationships that could be construed as a potential conflict of interest.
